# Trends in bio-behavioural risk factors of non-communicable diseases among adults in Sao Tome and Principe

**DOI:** 10.3389/fpubh.2023.1238348

**Published:** 2023-08-29

**Authors:** Supa Pengpid, Karl Peltzer

**Affiliations:** ^1^Department of Health Education and Behavioural Sciences, Faculty of Public Health, Mahidol University, Bangkok, Thailand; ^2^Department of Public Health, Sefako Makgatho Health Sciences University, Pretoria, South Africa; ^3^Department of Healthcare Administration, College of Medical and Health Science, Asia University, Taichung, Taiwan; ^4^Department of Psychology, University of the Free State, Bloemfontein, South Africa; ^5^Department of Psychology, College of Medical and Health Science, Asia University, Taichung, Taiwan

**Keywords:** noncommunicable disease, risk factors, trends, Sao Tome and Principe, STEPS survey

## Abstract

**Background:**

Understanding national trends in risk factors of noncommunicable diseases (NCDs) may have health policy implications. The aim of the study was to assess the prevalence and social and demographic factors associated with risk factors of NCDs in adults from 2008 to 2019 in Sao Tome and Principe.

**Methods:**

In repeat cross-sectional national STEPS surveys 2,457 adults (median age 37 years) in 2008 and 1,893 adults (median age 38 years) in 2019 in Sao Tome and Principe responded to structured interviews, physical and biochemical measures. Logistic regressions were applied to estimate predictors of NCD risk factors.

**Results:**

Having three to seven NCD risk factors significantly decreased among men but not women from 36.6% in 2008 to 26.8% in 2019. The proportion of specific risk factors of NCD increased significantly for low physical activity from 17.4% in 2008 to 30.9% in 2019, and overweight/obesity from 37.3% in 2008 to 51.0% in 2019. Insufficient fruit/vegetable consumption decreased from 83.1% in 2008 to 53.3% in 2019, frequent alcohol use from 32.6% in 2008 to 24.8% in 2019, and diabetes from 3.1% in 2008 to 1.2% in 2019, while the proportion of current tobacco use and hypertension remained unchanged from 2008 to 2019. Men engaged more often than women in current tobacco use and frequent alcohol use, and women had higher rates of low physical activity and overweight/obesity than men. Higher educational levels were positively associated with overweight/obesity, and inversely associated with frequent alcohol use and inadequate fruit/vegetable intake.

**Conclusion:**

Between 2008 and 2019, the prevalence of seven risk factors for NCDs in Sao Tome and Principe declined among men, but not among women. Several associated variables have been identified for each individual risk factor of NCD that may help guide interventions.

## Introduction

Most deaths from non-communicable diseases (NCDs) occur in lower resourced countries (>85%) (1). In the lower-middle-income island of Central Africa, Sao Tome and Principe, 55% of people died from NCDs in 2016 (2). Cardiovascular disease, diabetes, cancer, and respiratory disease, account for more than 80% of NCD premature deaths (1). In the general population of Sao Tome and Principe, 20% had high blood pressure and 6% hypoglycaemia (2). The ill-healthy diet, tobacco, alcohol consumption and low physical activity increase the risk of death from NCDs (1). In Sao Tome and Principe, 7% of adults engaged in harmful alcohol use and 15% were physically inactive (2). Among recent mothers in Sao Tome and Principe, more than 30% were overweight or obese (3). As the number of NCDs grows rapidly in the sub-Saharan region of Africa, including in Sao Tome and Principe (4), due to multiple factors, such as the adoption of an unhealthy lifestyle, and the increase in the average life expectancy of the population (4), it is important to understand the local factors affecting NCDs to monitor changes in burden, measure the progress of the NCD programme, and plan new activities (5). In this regard, data from national communities on single and multiple risk factors for NCDs among adults in Sao Tome and Principe are needed in planning health programmes.

In another island country in Africa in Comoros, among adults (25–64 years) the prevalence of diabetes was 8.5% (6), and overweight and obesity was 28.6 and 22.2%, respectively (7). In other African countries, for example, in Malawi, 16.5% had 3–7 risk factors of NCD (ranging from 5.6% raised blood glucose to 32.9% raised blood pressure) (8), and in Zambia, 26.7% had 3–10 risk factors of NCD (ranging from 6.2% diabetes to 90.4% insufficient fruit and vegetable intake = IFVI) (9).

Social and demographic factors associated with NCD risk factors include older age (10–13), male sex (10, 14), educational level (10, 14), and residing in urban areas (13, 14). The aim of the investigation was to estimate the prevalence and sociodemographic factors associated with seven risk factors of NCDs in adults from 2008 to 2019 in Sao Tome and Principe.

## Methods

Secondary data from two cross-sectional STEPS surveys conducted in 2008 and 2019 in Sao Tome and Principe were analyzed, with a total response rate of 95.5% in 2008 and 91.5 percent in 2019 (15). Multi-step stratified sampling was conducted to select one person at random of the target age (25–64 years in 2008, and 18–69 years in 2019; the 2019 survey was restricted to 25–64 years for this analysis) per household (15).

SaoTome and Principe has a population of 228,000 in 2022 (16), a literacy rate of 92.8%, an urbanization rate of 76.4% (17), a gross domestic product (GDP) *per capita* in 2019 of $ 1961; the life expectancy (at birth) increased from 63.5 years in 2000 to 70.4 years in 2019 (18). The NCD programme in Sao Tome and Principe includes implementing the NCD programme and promotion of healthy lifestyles (19). Regarding integration of the NCD programme into primary health care (PHC) in Sao Tome and Principe, partial implementation includes having NCD guidelines, generally available medicine, generally available technologies, and drug therapy at PHC, and not implemented a national list of essential medicines and technology (20), and adhering to the WHO Package of Essential NCDs (PEN) interventions (21). Acccording to the WHO NCD monitor (22), there is poor implementation of NCD reduction measures, including unhealthy diet, tobacco demand and harmful use of alcohol, and public education and awareness campaigns on physical activity.

Study approval was provide by the Ministry of Health Ethical Committee, Sao Tome and Principe, and participants gave written informed consent (15). The data collection followed the WHO’s three STEPS methodology: the first step included the administration of questionnaires (social epidemiology, medical history, use of medicines and health risk behaviour); the second step involved body weight, height and blood pressure measurements; and the third step included biochemical tests (glucose and blood lipids) (23).

### Assessments

*Risk factors for NCDs* were included on the basis of previous research (8, 10, 11, 24), as follows: *Behavioural risk factors of NCD* included current tobacco use, frequent alcohol use (≥5 days/week) (15), low physical activity defined by the “Global Physical Activity Questionnaire” (25), and IFVI (<5 servings/day); *Biological risk factors for NCD:* Diabetes: fasting plasma glucose ≥7.0 mmol/L, and/or presently medication for diabetes (26); Hypertension: measured blood pressure (BP) (average of two of last three measurements) defined as “systolic BP ≥140 mm Hg and/or diastolic BP ≥90 mm Hg” or presently on medication for hypertension (27); measured body mass index (overweight: 25.0–29.9 kg/m^2^ and obesity: ≥30.0 kg/m^2^) (26).

*Social and demographic information* consisted of sex, age, education, adult members in household and interview language (15).

### Statistical analysis

The proportion of NCD risk factors was grouped on the basis of previous studies (13, 24), 3–7 NCD risk factors (compared to 0–2 risk factors). Chi-square tests were used to estimate differences in proportions. Logistic regressions were used to assess predictors of each of the seven NCD risk factors, adjusted by study year, proportion of adult members, education, sex, age, and interview language. Missing values (IFVI = 5.3%, low physical activity = 1.5%, current tobacco use = 0.1%, frequent alcohol use = 0%, overweight/obesity = 2.1%, hypertension = 2.0%, and diabetes = 7.1%) were discarded and *p* < 0.05 indicated significance. Statistical analyses were done with STATA software version 15.0 accounting for sample weighting and complex study design. The analysis weight was calculated by taking the reverse probability of each participant’s selection. These weights were adjusted to accommodate differences in the age-sex composition of the sample population compared to the target population. Different weight variables were calculated for (1) for interview data, (2) for physical measures, and (3) for biochemical measures (15).

## Results

### Sample characteristics

The sample included 2,457 adults, with a median age 37 years (IQR = 29–47) in 2008 and 1893 adults median age 38 years (IQR 32–48) in 2019. The proportion of younger adults increased, and higher education and number of adult household members decreased from 2008 to 2019 (see Table 1).

**Table 1:** Sociodemographic characteristics of individuals 25 to 64 years and older in Sao Tome and Principe 2008 and 2019.

Variable	Study year		*p*-value
	2008	2019	
	*N* = 2,457	*N* = 1893	
	*N* (%)	*N* (%)	
Age (years)			
25–34	976 (39.7)	614 (32.4)	<0.001
35–44	731 (29.8)	671 (35.4)	
45–64	750 (30.5)	608 (32.1)	
Gender			
Female	1,407 (57.3)	1,139 (60.2)	0.054
Male	1,050 (42.7)	754 (39.8)	
Education			
<primary	529 (21.6)	548 (28.9)	<0.001
primary	889 (36.3)	1,003 (53.0)	
>primary	1,028 (42.0)	342 (18.1)	
Adults household members			
1	465 (19.0)	757 (40.0)	<0.001
2	1898 (52.9)	879 (46.4)	
≥3	689 (28.1)	257 (13.6)	
Interview language			
Other	125 (5.1)	74 (3.9)	0.482
Portuguese	2,332 (94.9)	1819 (96.1)	

Unweighted percent.

### Distribution of NCD risk factors from 2008 to 2019

The proportion of specific risk factors of NCD risk factors increased significantly for low physical activity from 17.4% in 2008 to 30.9% in 2019, and overweight/obesity from 37.3% in 2008 to 51.0% in 2019. There was a significant decrease in IFVI from 83.1% in 2008 to 53.3% in 2019, frequent alcohol use from 32.6% in 2008 to 24.8% in 2019, and diabetes from 3.1% in 2008 to 1.2% in 2019, while the prevalence of current tobacco use and hypertension remained unchanged from 2008 to 2019. Among men but not among women, current tobacco use and frequent alcohol use significantly decreased from 2008 to 2019, while among women but not among men diabetes significantly decreased. The overweight/obesity prevalence only increased significantly among women (from 43.3 to 58.1%) but not among men. Having 3–7 NCD risk factors decreased from 36.6% in 2008 to 31.7% in 2019, but this was only significant for men and not for women and overall (see Table 2).

**Table 2:** Prevalence of risk factors for non-communicable diseases (NCDs) among adults in Sao Tome and Principe 2008 and 2019.

NCD risk factors	2008	2019	*p*-value
	N (% 95 CI)	N (% 95 CI)	
All	2,457	1893	
Fruit/vegetable intake (<5 servings/day)	1936 (83.1, 81.5–84.7)	928 (53.3, 46.2–60.2)	<0.001
Low physical activity	427 (17.4, 15.4–19.5)	539 (30.9, 26.2–36.0)	<0.001
Current tobacco use	205 (9.0, 8.0–10.1)	189 (8.8, 7.5–10.4)	0.860
Frequent alcohol use	781 (32.6, 30.1–35.3)	533 (24.8, 21.8–28.1)	<0.001
General overweight/obesity	929 (37.3, 32.1–42.8)	891 (51.0, 48.3–53.8)	<0.001
Hypertension	825 (35.0, 31.8–38.4)	618 (33.5, 30.7–36.3)	0.465
Diabetes	76 (3.1, 2.4–3.9)	21 (1.2, 0.8–2.0)	<0.001
3–7 NCD risk factors	753 (36.3, 31.4–41.5)	452 (31.7, 27.8–35.9)	0.157
Male	1,050	754	
Fruit/vegetable intake (<5 servings/day)	819 (83.3, 81.6–84.8)	370 (52.5, 44.3–60.5)	<0.001
Low physical activity	100 (10.0, 6.6–15.0)	167 (24.0, 19.4–29.3)	<0.001
Current tobacco use	144 (14.0, 12.5–15.9)	131 (18.0, 15.2–21.3)	0.024
Frequent alcohol use	449 (42.6, 38.7–46.7)	264 (32.2, 28.0–36.8)	<0.001
General overweight/obesity	317 (30.6, 24.8–37.0)	281 (35.4, 31.3–39.8)	0.209
Hypertension	354 (36.1, 32.6–39.8)	226 (31.4, 27.0–36.2)	0.121
Diabetes	25 (2.5, 2.0–3.1)	10 (1.5, 0.7–3.4)	0.252
3–7 NCD risk factors	330 (36.6, 29.8–43.9)	161 (26.8, 21.9–32.2)	0.026
Female	1,407	1,139	
Fruit/vegetable intake (<5 servings/day)	1,117 (83.0, 80.7–85.0)	558 (53.6, 46.2–60.9)	<0.001
Low physical activity	327 (23.9, 22.5–25.2)	372 (33.9, 28.6–39.5)	<0.001
Current tobacco use	61 (4.4, 3.0–6.4)	58 (4.9, 3.5–6.8)	0.721
Frequent alcohol use	232 (23.6, 21.0–26.4)	269 (21.6, 18.2–25.4)	0.342
General overweight/obesity	612 (43.3, 38.4–48.4)	610 (58.1, 54.4–61.6)	<0.001
Hypertension	471 (34.1, 30.1–38.2)	392 (34.3, 30.9–37.9)	0.922
Diabetes	51 (3.6, 2.7–4.8)	11 (1.1, 0.6–2.1)	<0.001
3–7 NCD risk factors	465 (36.1, 32.6–39.6)	291 (33.7, 29.0–38.8)	0.445

CI, confidence interval.

### Social and demographic associations with individual behavioural risk factors for NCDs

Compared to participants in the 2008 study, participants in the 2019 study had a significantly higher prevalence of low physical activity, significantly lower IFVI and fewer frequent alcohol consumption. Older groups were more likely to use tobacco at present, and middle-aged participants were more likely to consume alcohol frequently. Men engaged more often than women in current tobacco use and frequent alcohol use, and women had higher rates of low physical activity and than men. Higher educational levels were negatively associated with IFVI, and frequent alcohol use. Having two or three or more adult household members (lower economic status) was negatively associated with low physical activity and IFVI. Portuguese interview language increased the odds of frequent alcohol use, low physical activity and IFVI (see Table 3).

**Table 3:** Determinants of behavioural non-communicable disease risk factors in Sao Tome and Principe 2008 and 2019.

Variable	Inadequate fruit/vegetable intake	Low physical activity	Current tobacco use	Frequent alcohol use
	AOR (95% CI)	AOR (95% CI)	AOR (95% CI)	AOR (95% CI)
Study year				
2008	1 (Reference)	1 (Reference)	1 (Reference)	1 (Reference)
2019	0.17 (0.12 to 0.24)***	1.82 (1.46 to 2.28)***	1.15 (0.86 to 1.54)	0.53 (0.41 to 0.68)***
Age (years)				
25–34	1 (Reference)	1 (Reference)	1 (Reference)	1 (Reference)
35–44	0.90 (0.68 to 1.18)	0.95 (0.69 to 1.31)	1.19 (0.70 to 2.03)	1.39 (1.08 to 1.81)*
45–64	1.09 (0.78 to 1.50)	1.17 (0.89 to 1.55)	1.84 (1.08 to 3.12)*	1.05 (0.77 to 1.43)
Gender				
Female	1 (Reference)	1 (Reference)	1 (Reference)	1 (Reference)
Male	1.02 (0.79 to 1.33)	0.59 (0.47 to 0.75)***	4.67 (2.99 to 7.29)***	1.95 (1.52 to 2.49)***
Education				
<primary	1 (Reference)	1 (Reference)	1 (Reference)	1 (Reference)
primary	0.88 (0.64 to 1.21)	0.92 (0.66 to 1.28)	0.78 (0.51 to 1.17)	0.66 (0.48 to 0.89)**
>primary	0.53 (0.33 to 0.84)**	1.36 (0.91 to 2.01)	0.65 (0.34 to 1.25)	0.30 (0.19 to 0.46)***
Adult members				
1	1 (Reference)	1 (Reference)	1 (Reference)	1 (Reference)
2	0.70 (0.54 to 0.91)**	0.76 (0.58 to 0.99)*	0.78 (0.53 to 1.15)	0.85 (0.65 to 1.12)
≥3	0.71 (0.49 to 1.05)	0.51 (0.32 to 0.79)**	0.91 (0.50 to 1.66)	0.65 (0.42 to 1.01)
Interview language				
Other	1 (Reference)	1 (Reference)	1 (Reference)	1 (Reference)
Portuguese	4.56 (2.31 to 8.99)***	4.32 (1.43 to 13.04)*	3.29 (0.63 to 17.05)	2.57 (1.15 to 5.72)*

AOR, adjusted odds ratio; *p < 0.05, **p < 0.01, ***p < 0.001.

### Sociodemographic determinants of individual biological NCD risk factors

Compared to 2008 participants, 2019 participants had a significantly higher prevalence of overweight/obesity and a lower prevalence of diabetes. Age was positively associated with obesity/obesity, hypertension and diabetes. Women had a higher rate of overweight/obesity than men. Higher educational levels and Portuguese interview language were associated with overweight/obesity. The number of adult households did not affect the prevalence of three biological risk factors for NCDs (see Table 4).

**Table 4:** Asociations with biological non-communicable disease risk factors in Sao Tome and Principe 2008 and 2019.

Variable	General overweight/obesity	Hypertension	Diabetes
	AOR (95% CI)	AOR (95% CI)	AOR (95% CI)
Study year			
2008	1 (Reference)	1 (Reference)	1 (Reference)
2019	1.86 (1.44 to 2.39)***	0.87 (0.64 to 1.18)	0.42 (0.20 to 0.89)*
Age (years)			
25–34	1 (Reference)	1 (Reference)	1 (Reference)
35–44	1.99 (1.53 to 2.60)***	2.24 (1.67 to 3.00)***	4.21 (1.13 to 15.63)*
45–64	1.87 (1.47 to 2.38)***	5.12 (3.75 to 7.01)***	14.46 (2.80 to 74.61)**
Gender			
Female	1 (Reference)	1 (Reference)	1 (Reference)
Male	0.34 (0.27 to 0.43)***	0.91 (0.69 to 1.20)	1.29 (0.46 to 3.59)
Education			
<primary	1 (Reference)	1 (Reference)	1 (Reference)
primary	1.28 (1.00 to 1.64)	0.81 (0.62 to 1.05)	1.18 (0.37 to 3.82)
>primary	2.32 (1.62 to 3.33)***	0.77 (0.51 to 1.16)	2.18 (0.48 to 10.04)
Adults household members			
1	1 (Reference)	1 (Reference)	1 (Reference)
2	1.24 (0.97 to 1.57)	0.97 (0.71 to 1.33)	0.75 (0.26 to 2.19)
≥3	1.15 (0.78 to 1.71)	0.83 (0.57 to 1.21)	0.42 (0.08 to 2.21)
Interview language			
Other	1 (Reference)	1 (Reference)	1 (Reference)
Portuguese	2.43 (1.48 to 4.00)***	1.58 (0.87 to 2.86)	0.91 (0.11 to 7.68)

AOR, adjusted odds ratio; *p < 0.05, **p < 0.01, ***p < 0.001.

## Discussion

The study showed that having 3–7 NCD risk factors among male but not female adults significantly decreased from 36.6% in 2008 to 26.8% in 2019 in Sao Tome and Principe. Some of this reduction may be attributed to strengthened implementation of NCD programme in Sao Tome and Principe (19). Having overweight/obesity and low physical activity significantly increased, and IFVI, frequent alcohol use, and diabetes significantly decreased from 2008 to 2019, while the prevalence of current tobacco use and hypertension remained unchanged from 2008 to 2019. According to the Global Nutrition Report (28), Sao Tome and Principe did not achieve their diet NCD targets, with still having high rates of obesity (19.7% in women and 8.9% in men). Similar to previous trend studies in India (29), Mongolia (30, 31) and Iran (32) having high body mass index and/or low physical activity increased over time. Although Sao Tome has implemented some national food and NCD policies, such as sugar-sweetened beverage tax and operational policy, strategy, or action plan to reduce unhealthy diet related to NCDs, additional implementation of some of the following are indicated food-based dietary guidelines, policy to eliminate industrially produced trans fatty acids, policy to reduce the impact of marketing of foods and beverages high in saturated fats, trans fatty acids, free sugars, or salt on children, and policy to limit saturated fatty acid intake (28). Regarding physical inactivity, the implementation of some of the following may help in increasing physical activity, including national physical activity communications campaigns, national mass participation events on physical activity, promotion of physical activity in different settings, brief intervention on physical activity in primary health care, quality physical education and national guidelines on physical activity in different age groups (16).

In this survey the prevalence of diabetes reduced over time, whereas in India (29), South Kivu, Democratic Republic of Congo (33), and Mozambique (34), the prevalence of diabetes increased over time. The unchanged current tobacco use in Sao Tome and Principe may be attributed to reduced compliance with the MPOWER tobacco control strategy, e.g., cigarettes have not become less affordable, no smokefree laws in public places, no tollfree telephone quit line, no smoking cessation support available in the community and health care setting, and no anti-tobacco mass media campaigns (35).

Furthermore, frequent alcohol use was significantly reduced from 2008 to 2019 in this study, while the recorded alcohol *per capita* (15+) consumption also reduced from 8.3 in litres of pure alcohol in both sexes in 2010 to 6.8 in 2016 (36). Compared to other African countries, having 3–7 risk factors for NCDs in this study (36.3% in 2008 and 31.7% in 2019) was higher than in Malawi (3–7 risk factors, 16.5%) (8) and Zambia (26.7%, 3–10 risk factors) (9). The prevalence of overweight/obesity in 2019 (51.0%) and the prevalence of diabetes (1.2%) in 2019 was similar or lower than in another island country in Africa in Comoros (50.8% overweight/obesity and 8.5% diabetes) (6, 7), but in terms of overweight/obesity higher than in Malawi (26.5%) (8) and Zambia (24.4%) (9). The prevalence of hypertension (33.5% in 2019) was similar to Malawi (32.9%) (8) but higher than in Zambia (18.9%) (9), the prevalence of current tobacco use (8.8% in 2019) was lower than in Malawi (14.1% current smokers) (8), and in Zambia (10.7% daily tobacco use) (9), and the rate of IFVI (53.3% in 2019) was lower than in Zambia (90.4%) (9).

There was a male preponderance of current tobacco use and frequent alcohol use and a female preponderance of general overweight/obesity and low physical activity, and there were no sex differences in hypertension, diabetes and IFVI. In previous studies (11, 24), the prevalence of substance use in men was higher than in women, and the rate of obesity/obesity in women was higher than in men. As expected (10–13), older age increased the odds of overweight/obesity, hypertension, diabetes, and current tobacco use and middle age was associated with frequent alcohol use. Lower education was associated with IFVI as well as with frequent use of alcohol and inversely associated with overweight/obesity. Portuguese interview language increased the odds of IFVI, low physical activity, frequent alcohol use and overweight/obesity. These findings show that the seven specific risk factors for NCDs can be targeted differently according to gender, age, level of education and interview language.

Comprehensive interventions can be directed to promote the control of body weight, the cessation of smoking, healthy diet, and the examination and control of high levels of sugar and blood pressure.

### Study strength and limitations

The STEPS surveys in Sao Tome and Principe utilized nationally representative data and standardised assessment tools. The two surveys conducted were cross-sectional, thus hindering causative conclusions. Some variables, such as substance use, were assessed by self-report, which may have biased responses. The Sao Tome and Principe 2008 STEPS survey did not measure heavy drinking, other alcohol measures, household income and residence status, which could therefore not be included in the analysis. The total cholesterol variable was excluded from the analysis because of implausible values in 2019 (77.8% had elevated total cholesterol).

## Conclusion

Based on two national household surveys in adults from 2008 to 2019 in Sao Tome and Principe, we found that the prevalence of seven risk factors of NCD decreased among men but not women from 2008 to 2019. Overweight/obesity, and low physical activity increased, and IFVI, frequent alcohol use, and diabetes decreased, and hypertension and current tobacco use stayed the same. Several factors associated with the risk factors of NCD have been identified, including age, gender, level of education and interview language that can guide interventions.

## Data availability statement

Publicly available datasets were analyzed in this study. This data can be found here: World Health Organization NCD Microdata Repository at https://extranet.who.int/ncdsmicrodata/index.php/catalog/893.

## Ethics statement

The studies involving humans were approved by Ministry of Health Ethical Committee, Sao Tome and Principe. The studies were conducted in accordance with the local legislation and institutional requirements. The participants provided their written informed consent to participate in this study.

## Author contributions

SP and KP fulfil the criteria for authorship, conceived and designed the research, performed statistical analysis, drafted the manuscript, and made critical revisions of the manuscript for key intellectual content. All authors contributed to the article and approved the submitted version.

## Conflict of interest

The authors declare that the research was conducted in the absence of any commercial or financial relationships that could be construed as a potential conflict of interest.

## Publisher’s note

All claims expressed in this article are solely those of the authors and do not necessarily represent those of their affiliated organizations, or those of the publisher, the editors and the reviewers. Any product that may be evaluated in this article, or claim that may be made by its manufacturer, is not guaranteed or endorsed by the publisher.
